# Modeling the effect of environmental cytokines, nutrient conditions and hypoxia on CD4^+^ T cell differentiation

**DOI:** 10.3389/fimmu.2022.962175

**Published:** 2022-09-23

**Authors:** David Martínez-Méndez, Leonor Huerta, Carlos Villarreal

**Affiliations:** ^1^ Instituto de Física, Universidad Nacional Autónoma de México, Mexico City, Mexico; ^2^ Instituto de Investigaciones Biomédicas, Departamento de Inmunología, Universidad Nacional Autónoma de México, Mexico City, Mexico

**Keywords:** CD4^+^ T cells, lymphocytes, mathematical model, metabolism, nutrients, hypoxia, hybrid phenotypes

## Abstract

Upon antigen stimulation and co-stimulation, CD4^+^ T lymphocytes produce soluble factors that promote the activity of other immune cells against pathogens or modified tissues; this task must be performed in presence of a variety of environmental cytokines, nutrient, and oxygen conditions, which necessarily impact T cell function. The complexity of the early intracellular processes taking place upon lymphocyte stimulation is addressed by means of a mathematical model based on a network that integrates variable microenvironmental conditions with intracellular activating, regulatory, and metabolic signals. Besides the phenotype subsets considered in previous works (Th1, Th2, Th17, and Treg) the model includes the main early events in differentiation to the T_*FH*_ phenotype. The model describes how cytokines, nutrients and oxygen availability regulate the differentiation of naïve CD4^+^ T cells into distinct subsets. Particularly, it shows that elevated amounts of an all-type mixture of effector cytokines under optimal nutrient and oxygen availability conduces the system towards a highly-polarized Th1 or Th2 state, while reduced cytokine levels allow the expression of the Th17, Treg or T_*FH*_ subsets, or even hybrid phenotypes. On the other hand, optimal levels of an all-type cytokine mixture in combination with glutamine or tryptophan restriction implies a shift from Th1 to Th2 expression, while decreased levels of the Th2-inducing cytokine IL-4 leads to the rupture of the Th1-Th2 axis, allowing the manifestation of different (or hybrid) subsets. Modeling proposes that, even under reduced levels of pro-inflammatory cytokines, the sole action of hypoxia boost Th17 expression.

## 1 Introduction

Antigenic stimulation and costimulation of naïve CD4^+^ T cells leads to their activation and differentiation towards effector cells that play important roles in adaptive immunity. Differentiation is strongly influenced by particular exogenous cytokines present in the microenvironment. The activation process also involves the activity of metabolic mediators necessary to fulfill the bioenergetic and biosynthetic demands of cell proliferation and function. CD4^+^ T cells transit from the catabolic state displayed by resting naïve and memory T cells, to an anabolic state necessary for growth and proliferation of effector T cells (1). Furthermore, as T cells differentiate during the immune response, they migrate from nutrient-replete lymphoid organs to sites of infection with limited amounts of nutrients and oxygen (1).

Each T cell effector subset is induced by different cytokines that activate a program involving the expression of lineage-defining transcription factors which induce the production of select cytokines and chemokine receptors to best control specific pathogens or prevent immune pathologies (reviewed in (2). Th1 cells are induced by IL-12, IL-18, and IFN-*γ* , express the T-bet transcription factor and produce IFN-*γ* . Th2 cells require IL-4 and are stabilized by IL-2, express GATA3 and produce IL-4, IL-5, and IL-13. Recently, IL-33 has emerged as a crucial immune modulator with pleiotropic activities in Th1 and Th2 immune responses (3). Th17 cells require TGF-*β* and IL-6, IL-21, or IL-23, express ROR*γ*t, and produce IL-21, IL-17A, and IL-17F. Treg cells require TGF-*β* and IL-2, express Foxp3, TGF-*β* and IL-10 in some cases. T_*FH*_ cells express the Bcl-6 transcription factor and produce IL-21 and IL-9 (4–7). Recently, it has been uncovered the capacity of polarized T cells to change their phenotype and repolarize towards mixed or alternative fates, particularly of the Th17 and pTreg cell subsets, implying that CD4^+^ T cells are adaptable and can exhibit phenotypic plasticity in response to changing contexts (2). Growing evidence indicates that nutrient and oxygen availability may alter the cellular fate normally associated to a specific pattern of exogenous cytokines. It has been shown, for example, that glutamine-deprived naive CD4^+^ T cells differentiate into Treg cells, even in microenvironment conditions that normally induce Th1 cells (8).

In this framework, the analysis of CD4^+^ T-cell function based on continuous logic networks constitutes an ideal tool to get an integrated view of the early steps by which these cells adjust their function to variable environmental conditions, including metabolic reprogramming. We previously assessed the effect of relative concentrations and combinations of exogenous cytokines on the expression levels of transcription factors in CD4 ^+^ T cell differentiation. With that purpose, we implemented a continuous regulatory network strictly complying cytokines and transcription factors leading to expression of Th1, Th2, Th17, Treg, Tr1, Th3, and T_*FH*_ phenotypes. The model allowed the construction of phenotypic space diagrams illustrating polarization changes depending on critical concentrations of environmental cytokines (9). In subsequent works, we put forth much broader schemes by use of regulatory networks with interactive rules defining intracellular signaling pathways involved in the reinforcement, diversification, and regulation of the initial antigenic and co-stimulatory signals, both in the Boolean (10) and continuous logic approaches (11). In the latter case, the model was able to delineate the temporal evolution of key events taking place after T-cell receptor (TCR) and CD28 stimulation: expression of activation transcription factors (NFkB, N-FAT, and AP-1), induction of the IL-2 cytokine and its feedback effect, the course of oxidative phosphorylation and glycolysis, the induction of anergy in the absence of costimulation through the activity of the anergy-inducing NDRG1 protein, the checkpoint blockade induced by CTLA-4, and differentiation to the Th1, Th2, Th17, and Treg effector phenotypes.

**Figure 1:**
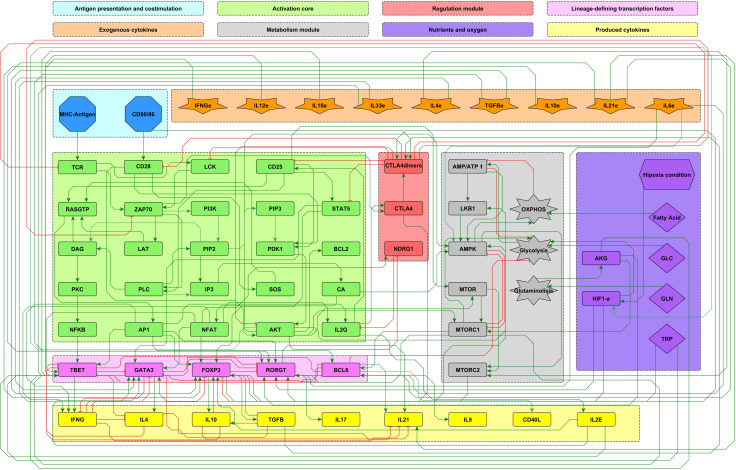
Modular 68-node network of early events in CD4^+^ T cell activation. Modules are described in the upper panel and their respective elements are indicated with the corresponding color in the network. Three modules correspond to inputs or entries of the system: antigen presentation and co-stimulation (blue), microenvironmental cytokines (orange), and nutrient and oxygen availability (purple). Four modules describe the intracellular interactions leading to activation and production of cytokines: an activation core (green), metabolic regulation including AMPK as the central regulator (grey), a regulatory module including the activity of CTLA-4 and the anergy factor NDRG1 (red), and the lineage-defining transcription factors leading to the production of specific cytokines defining the effector phenotypes Th1, Th2, Th17, T_*FH*_ and Treg (pink). Finally, another module includes the output cytokines produced by differentiated cells (yellow). Green and red edges represent activator and inhibitory pathways, respectively. The set of interacting fuzzy logic rules defining the dynamical system is shown in **Supplementary Material 1**. Construction of the network can be consulted in (10). The complete set of ordinary differential equations is provided in **Supplementary Material 2**. An interactive network file can be consulted online in https://bit.ly/3ar1Ojp. AKG, *α*-keto glutarate; GLC, glucose; GLN, glutamine; TRP, tryptophan; HIF1-*α*, hypoxia-inducible factor 1-alpha.

The analysis of the function of the CD4^+^ T cells based on continuous logic networks provides an outline of the way in which cells may adjust the function of its components to variable physiological or pathological environmental conditions associated to variable concentrations of phenotype-inducing cytokines, nutrients, and oxygen. With this purpose, the set of regulatory interactions considered in a previous work (11) has been extended to include the induction of the T_*FH*_ effector phenotype, conducing to a regulatory network composed of 68 nodes which is presented as a modular scheme **(** **Figure 1** **)**, facilitating the identification of every node’s role.

## 2 Methods

### 2.1 Network inference

#### 2.1.1 Network modular organization

The development of the current model of T cell function has involved a sequential approach based on the integration of modules with diverse degree of complexity, starting from a minimal regulatory network of the differentiation process (9), continuing with those associated to early activation steps (10), and followed by the elements participating in the metabolic regulation of cell differentiation (11). For the construction of the present network, we considered that, along with cytokines, nutrients and hypoxia are prime exogenous agents influencing T cell function.

**Figure 2:**
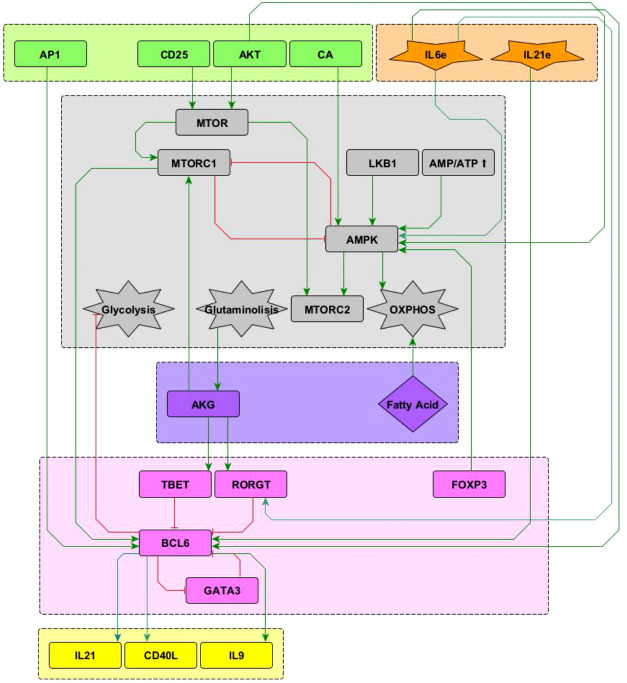
Interactions of the Bcl-6 lineage-defining transcription factor (shown in pink) included in the network. See “Network inference” section for description. Subnetworks containing other interactions of the T-bet, ROR*γ* t and GATA3 nodes can be seen in Martinez2020.

**Figure 3:**
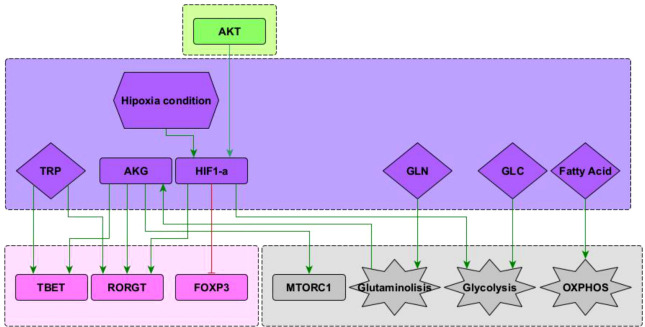
Subnetwork showing interaction of the nodes representing HIF1-*α* , glutamine (GLN), glucose (GLU), and fatty acids included in the network. See “Network inference” section for description. Subnetworks for other interactions of T-bet, ROR*γ* T and Foxp3 nodes can be seen in (10). Subnetworks for other interactions of the mTORC1, glycolysis and OXPHOS nodes can be seen in Martínez-Méndez2021.

The present model incorporates the induction of the *T*_*FH*_ effector phenotype during the dendritic cell (DC) phase, as well as interactions associated to microenvironmental nutrients (glutamine and tryptophan), and hypoxia. The network involves 68 nodes organized in eight modules (**Figure 1**). Three modules correspond to inputs or entries of the system: 1) antigen presentation and co-stimulation, 2) phenotype-inducing microenvironmental cytokines, and 3) nutrient and oxygen availability; four modules represent intracellular interactions leading to activation and production of cytokines, including 4) an activation core containing nodes located downstream TCR and CD28 and involving pathways leading to production of IL-2, with expression of its high affinity receptor, CD25, which mediates cytokine effects; 5) a metabolic regulation module with the nutrient sensor, AMPK, acting as the central regulator of glycolysis and OXPHOS; 6) a regulatory module including the activity of CTLA-4 and the anergy factor NDRG1; 7) a module corresponding to the expression of the the lineage-defining transcription factors T-bet, GATA3, Foxp3, ROR*γ*t and Bcl-6; 8) a module including the output cytokines produced by differentiated cells. The modular organization of the network allows the rapid identification of the node’s main role, as well as the introduction of new nodes as part of functional modules. The module representing interactions leading to differentiation of the *T*_*FH*_ phenotype in the DC phase is presented in **Figure 2**, while the action of nutrients and hypoxia is represented in **Figure 3**.

#### 2.1.2 T_FH_ polarization

Interactions of the node representing Bcl-6, the lineage-defining transcription factor of T_*FH*_ cells (12) with other elements in the network, is shown in **Figure 2**. T_*FH*_ cells are induced by a strong TCR signal from MHC-peptide complexes expressed on antigen presenting cells (APC) in the presence of IL-6 and IL-21. The downstream activation of Bcl-6 *via* mTORC1 (13, 14) and AP-1 (15) promotes T_*FH*_ cell differentiation. It is known that the transcription factors T-bet and ROR*γ*t inhibit the activity of Bcl-6 [reviewed in (12, 16–18)]. It has been proposed that GATA-3 may inhibit the activity of Bcl-6 (19); to test this proposal this interaction has been included in the network. On the other hand, Bcl-6 directly represses the gene program of the glycolysis pathway, thus playing an important metabolic role (20). Concomitantly, IL-6 induces activation of the metabolic regulator AMP-activated protein kinase (AMPK), favoring a metabolism based on oxidative phosphorylation (21, 22). Notably, IL-6 activates both the ROR-*γ*t and Bcl-6 transcription factors, inducing polarization of effector cells towards the Th17 and T_*FH*_ phenotypes. In this scenario, it has been observed that the presence of TGF-*β* promotes the expression of ROR-*γ*t, so that the absence of TGF-*β* allows the expression of Bcl-6 (23). Differentiated T_*FH*_ cells express the CXCR5 chemokine receptor, the CD40L molecule, and produce IL-21 and IL-9. The current network only describes the DC phase but not the germinal center phase of T_*FH*_ induction. So, events related to ICOS signaling, production of IL-4 and IFN-*γ* , and induction of unresponsiveness to IL-2 in germinal center were not included.

#### 2.1.3 Glucose, fatty acids, and glutamine in T CD4^+^ cell differentiation

Predominance of glycolysis or oxidative phosphorylation (OXPHOS) is closely linked to activation and function of CD4^+^ T cells. Microenvironmental glucose and fatty acids are internalized to feed these pathways. In addition, glutamine is another important biosynthetic precursor influencing cell differentiation **(** **Figure 3** **)**. Glutamine undergoes glutaminolysis and it is metabolized into *α* -keto glutarate (AKG), which enters the mitochondrial citric acid cycle. In addition, AKG upregulates mTORC1 and enhances glycolysis (8, 24). AKG is required for Th1, while blocking Treg cell differentiation in a mTORC1-dependent manner (25). Furthermore, AKG can shift the balance between Th1/Treg differentiation, since it promotes the expression of the transcription factor T-bet. Instead, activation of naïve CD4^+^ T cells under conditions of glutamine deprivation results in their differentiation into forkhead box P3-positive (Foxp3) regulatory T (Treg) cells (8). Recently, it has been observed that glutamine metabolism play a critical role in the balance between Th17/Treg differentiation favouring Th17 polarization, at least under standard cell culture conditions (26–28). These interactions have been included in **Figure 3**.

#### 2.1.4 Hypoxia

Hypoxia is common in proinflammatory environments where the differentiation of T cells is crucial to overcome an infection (29). Under low oxygen conditions, the hypoxia-inducible factor 1-alpha (HIF-1*α* ) is activated and influences the metabolism and differentiation of T cells. HIF-1*α* is positively regulated by PI3K–AKT–mTOR signals, particularly during persistent antigen stimulation along with an hypoxic environment. HIF-1*α* directly induces the expression of RORγt and thus supports the Th17 phenotype. In addition, it induces the expression of genes that are required for glycolysis, an activity that is directly opposed by Bcl-6 (20). It has been observed that HIF-1*α* can act as a metabolic switch. Under normoxic conditions HIF-1*α* is dowregulated in a oxygen dependent manner, involving the ubiquitin-proteasome pathway (30). Under low oxygen conditions HIF-1*α* is spared from degradation and translocates to the nucleus where it dimerizes and targets glycolysis, angiogenesis, and apoptosis genes (31). It has been suggested that HIF-1*α* can mediate the balance between TH17 and Treg differentiation. HIF-1*α* could directly activate the transcription factor ROR*γ*t thereby promoting a TH17 differentiation. Concurrently, HIF-1*α* can also attenuate the Treg differentiation by binding Foxp3 and targeting it for proteosomal degradation (32). These interactions are depicted in **Figure 3**.

#### 2.1.5 The role of tryptophan in T CD4^+^ cell differentiation

Tryptophan catabolism has emerged as a relevant regulatory pathway of the activity of T helper cells. Tryptophan is necessary for protein synthesis and proliferation of activated T cells. Degradation of tryptophan is a mechanism of tumor cell evasion of the innate and adaptive immune system. In addition, tryptophan has a relevant activity as activator of the ROR*γ*t and T-bet transcription factors and thus is able to induce polarization of CD4^+^ T cells to the Th17 and Th1 phenotypes (33, 34). Conversely, tryptophan catabolism induces a Treg phenotype [reviewed in (33)]. Indoleamine 2, 3-dioxygenases (IDO1 and IDO2) and tryptophan 2, 3-dioxygenase (TDO) are tryptophan catabolic enzymes that catalyze the conversion of tryptophan into kynurenine. IDO1 is overexpressed in the vast majority of cancers (35). Small-molecule inhibitors, such as epacadostat, have been developed to block IDO1 activity. In preclinical models, they can restore antitumoral T cell immunity and synergize with immune checkpoint inhibitors or cancer vaccines.

### 2.2 Fuzzy regulatory networks

In an effort to understand the gene regulatory processes involved in cellular development, C. H. Waddington introduced in 1957 the metaphoric concept of epigenetic landscape (36). He proposed a perspective of cellular development as a ball rolling down within a landscape formed by peaks and valleys. Following its trajectory, the ball may finally fall into a valley, representing its final position that defines a steady-state -and a cellular fate-, also known as attractor. Waddington’s epigenetic landscape was formalized, among others, by S. A. Kauffman, who studied the behavior of large networks of randomly interconnected binary “genes” with a dichotomous (on-off) behavior, establishing the principles of Boolean analysis in the modeling of regulatory networks in Biology (37).

Mathematical modeling based on Boolean networks provides meaningful qualitative information on the basic topology of relations that determine alternative cell fates and may be used for the analysis of biological circuits without requiring explicit values of the network parameters (38). In this description, the network nodes represent genes, transcription factors, proteins mediating signaling cascades, RNA, environmental factors, etc., while links represent activating or inhibitory regulations between pairs of nodes. This kind of approach yields important information on the regulation biological circuits, such as signalling cascades or switching modules. However, a more realistic description of biological systems requires the consideration that the expression levels, concentrations, and parameters of biological systems may acquire any value within a continuous range limited only by functionality constraints; therefore, a description merely based on a Boolean approach, although fundamental, is insufficient to account for the rich phenomenology observed in Nature and a less restrictive formalism must be contemplated. This may be achieved by introducing a continuous logical analysis (39, 40), whose early foundations were established by Glass and Kauffmann (41, 42).

In the present study we consider an alternative approach based on fuzzy logic (43, 44). Fuzzy logic is a theory originally aimed to provide formal foundation to imprecise or ambiguous statements in language theory; however, its predictive inference power has found applications in diverse disciplines, such as physical, biomedical, and control sciences. This formalism considers continuous fuzzy variables *p*,*q*,... with truth values ranging in the interval [0,1]. These variables fulfill the axioms of Boolean algebra, except for the principle of no contradiction (*q* and not *q*=0), or equivalently, the absence of the excluded middle ( *q* or not *q*=1 ). On these terms, the proposition *q*=not *q*, has a solution in fuzzy logic given by *q*=1/2; therefore, the parameter *θ*=*q*=1/2 represents a threshold between falsity and truth in fuzzy logic inferences. As a consequence, this theory admits the consideration of ‘half truths’, which in biological systems would imply, for example, the incomplete expression of a phenotype.

Fuzzy logic propositions can be built by introducing fuzzy logic connectives, equivalent of the Boolean operators ∧ (and), ∨ (or), ¬ (not). This may be performed in a variety of ways, denoted in the literature as t-norms. In this work we employ a multiplicative norm defined by


(1) p∧q→p·qp∨q→p+q−p·q¬q→1−q.

Using these rules, a fuzzy logic proposition, *v*_*k*_[*q*_1_,...,*q*_*n*_] , may be straightforwardly derived from its Boolean counterpart. For example, the Boolean proposition *q*_1_=*q*_1_∧(*q*_2_∨*q*_3_)∧(¬*q*_4_) is accordingly transformed into *v*_1_(*q*_1_,*q*_2_,*q*_3_,*q*_4_)=*q*_1_·(*q*_2_+*q*_3_−*q*_2_·*q*_3_)·(1−*q*_4_). Both statements define a logical inference implying that *q*_1_ is feedback-activated by *q*_1_ itself and, either *q*_2_ or *q*_3_ are activated, but *q*_4_ is inhibited. Clearly, the fuzzy expression is nonlinear, involving the cooperative action of network nodes.

The response provided by *v*_*k*_[*q*_1_,...,*q*_*n*_] involves an implicitly gradual character. This response may be translated into a categorical output, that is, one in which activation or inhibition may be clearly discerned, by introducing a characteristic function, *μ*[*v*_*k*_], defined in the interval [0,1] and showing a sigmoid-like behavior:


(2) $$\mu \left[v_{k}\right]=\frac{1}{1+e^{-\beta \left(v_{k}\left[q_{1},\text{...},q_{n}\right]-\theta \right)}}$$

Here, the parameter *β* represents the rate at which proposition *v*_*k*_ is expressed, and *θ*=1/2 is its threshold value. Here, we consider that *β*=10. Note that this procedure is similar to that employed in the transit from linear to logistic regression models for the probability of one event (out of two alternatives) to take place.

The interactive dynamics of the fuzzy network is now described by a set of ordinary differential equations:


(3) $$\frac{dq_{k}}{dt}=\mu \left[v_{k}\right]-\alpha_{k}q_{k}.$$

Where *μ*[*v*_*k*_] plays the role of an input, and *α*_*k*_ is the decay rate of node *k*. We observe that the steady state of the ODE system is determined by the condition *dq*_*k*_/*dt*=0, which conduces to


(4) $$q_{k}^{st}=\frac{1}{\alpha_{k}}\;\mu [v_{k}\left(q_{1}^{st},\text{...},q_{n}^{st}\right).$$

The former algorithmic procedure may be visualized as a formal realization of the epigenetic landscape metaphor, where the logical interactions define an underlying topography which may evolve accordingly with the network dynamics, until it finally attains a final steady configuration, being the valleys located at the sites defined by Eq.4. This expression shows that the equilibrium expression level of node *k* is affected, not only by the levels of other network nodes, but also by its own decay rate or, equivalently, by its characteristic expression time *τ*_*k*_=1/*α*_*k*_ . In this work we assume that the default value of the decay rates is *α*_*k*_=1 ; however, we observe that the topography of the landscape may be modified by alterations of the set {*α*_*k*_} , which acts as an ensemble of control parameters able to induce transitions between neighboring valleys in the landscape. This mechanism may be employed in the description plastic of phenotypic transitions (11). The methods for the construction of the fuzzy logic propositions are thoroughly described in the former reference.

#### 2.2.1 Modeling initial TCR and CD28 priming and CD28-CTLA-4 interactions

The intensity and time span of stimulation of CD4^+^ T cells due to MHC-antigen presentation to TCR and of CD80/86 binding to CD28 may be modified due to time-dependent variations in the number of TCR-MHC-peptide complexes, the presence of adhesion molecules, or TCR internalization or degradation after initial engagement, among other factors (4, 14, 45–49). The intensity of the TCR-MHC and CD28-CD80 engagement has been modeled by assuming that it occurs with an initial affinity strength, *A*_*MHC*_ and *A*_*CD*8086_, which is depleted by a factor, *D*_*MHC*_ and *D*_*CD*8086_, after a time lapse, *T*_*MHC*_ and *T*_*CD*8086_, respectively. This behavior was represented by step-like binding functions:


(5) $$MHC\left(t\right)=A_{MHC}-D_{MHC}/\left(1+exp\left[-\beta_{MHC}\left(t-T_{MHC}\right)\right]\right)$$

(6) $$CD8086\left(t\right)=A_{CD8086}-D_{CD8086}/\left(1+exp\left[-\beta_{CD8086}\left(t-T_{CD8086}\right)\right]\right)$$

Here, the parameter *β* denotes a saturation rate which we consider, for simplicity, *β*=1. In Ref (11). we have shown that a minimum stimulation time *T*=10 units is required to start the activation process; consistently, in this work we have assumed *T*_*MHC*_=*T*_*CD*8086_=15 time units. On the other hand, the parameter choice *A*_*MHC*_=*A*_*CD*8086_=1, *D*_*MHC*_=*D*_*CD*8086_=1 ensures a monotonous logistic behavior of the binding function.

In addition, in the description of T-cell priming interactions it must be taken into account the competitive action between CD28 and CTLA-4 for binding to CD80/86 since, upon activation, downstream interactions of CD28 with CTLA-4 may down-regulate the engagement of CD28 with CD80/86. The inhibitory capacity of CTLA4 is modeled, as in Ref. (11), by assuming that this is limited by its characteristic expression time, *T*_*CTLA*4_, which should be long enough to overwhelm the influence of factors that promote the transcription factors activity. In that reference we have shown that values of the parameter *T*_*CTLA*4_≤1 promote a sustained activation state before being arrested by the activity of CTLA-4.

### 2.3 Numerical methods

We coded a *Python* program to integrate the differential equation system and created an interactive interface to directly modify the initial conditions using the packages *numpy, scipy, matplotlib and ipywidgets*. For the computation, the differential equations system were solved for time intervals between *t*=0 to *t*≥30 using lsoda from the FORTRAN library “odepack” to obtain the function dynamics of each of the system components. The equations and python code used can be consulted in the GitHub repository “https://github.com/DrDavidMM/TCD4cell-activation-model-supplementary-material-.git”.

## 3 Results

### 3.1 Modeling activation and differentiation of CD4^+^ T cells to effector phenotypes

**Figure 4:**
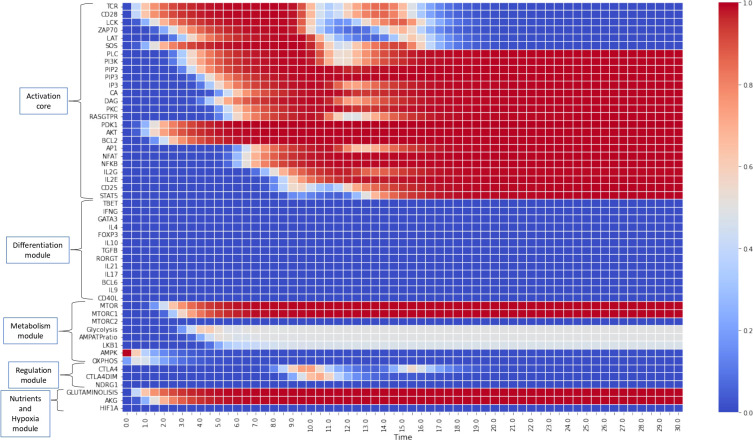
Heat map for the continuous dynamics of CD4^+^ T cell activation events in absence of exogenous cytokines (stimulation time *T*_*MHC*_=*T*_*CD*8086_=15 units). Colors indicate the expression level of each node, as shown in the right-hand column. Nodes are grouped into functional modules, whereas environmental input nodes are not shown. The initial network configuration is defined by optimal nutrient and normoxic conditions, as well as triggering induced by MHC-antigen and CD80/86. We observe that nodes between LCK and SOS are transiently expressed (according to the activation time) whereas the rest of nodes pertaining to the activation core remain stably activated. Concomitantly, the metabolic module (mTOR and mTORC1) is activated, leading to an initial boost of OXPHOS which decays giving place to a middle-level glycolytic metabolism.

**Figure 5:**
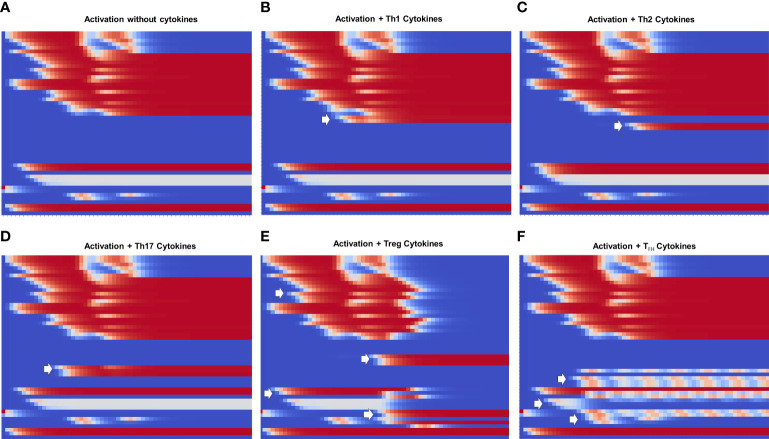
Heat map of the dynamics of CD4^+^ T cell activation and differentiation in the presence of exogenous cytokines inducing either Th1, Th2, Th17, Treg or T_*FH*_ phenotypes. The order of nodes and colors correspond to those shown in **Figure 4**. For comparison, we present activation in the absence of exogenous cytokines (**Figure A**). We observe that **(B–D)** Th1, Th2 and Th17 display the same glycolytic profile shown in **(A)**. In contrast, **(E)** Treg shows an initial transient glycolytic state, afterwards displaced by a high-level oxidative metabolism. **(F)** T_fh_ exhibits a periodic metabolic pattern alternating between OXPHOS and glycolysis. White arrows indicate activation of signatures specific for each phenotype: **(B)** T-bet and IFN-*γ* ; **(C)** GATA3 and IL-4, **(D)** ROR*γ*t, IL-17 and IL-21, **(E)** Foxp3, TGF-*β* and CTLA-4; **(F)** Bcl-6, CD40L, IL-21 and IL-9.

**Figures 4**, **5** show the dynamics of activation and differentiation as heat maps where the expression level of each node is indicated by its relative color brightness. The maps describe the time progression of the system from the naive to the differentiated stage. After initial triggering by interaction of MHC-peptide and CD80/86 with the TCR and CD28 molecules, the system evolves through signaling by downstream nodes, to a final steady state (attractor). Initial conditions were defined by sets of specific exogenous cytokines, and optimal nutrient and oxygen levels. Cell metabolism is characterized by an initial low level of oxidative phosphorylation along with AMPK activation.

**Figure 4** depicts the progression of activation in the absence of exogenous cytokines. TCR and CD28 are transiently expressed (in congruence with an activation time *T*_*MHC*_=*T*_8086_=15 units) in parallel with Lck and SOS, whereas the rest of nodes pertaining to the activation core become stably activated. This effect is related to the activity of the IL-2 feedback loop, which recurrently stimulates the system through the interaction of IL-2 with the inducible high affinity receptor subunit CD25 (10, 11). This loop maintains the system activation even after interruption of TCR and CD28 stimulation. Concomitantly, the metabolic module (mTOR and mTORC1) is activated, causing an initial gain of oxidative phosphorylation which subsequently decreases, giving way to a stable glycolytic metabolism, as shown before (11). Sustained activation is consistent with a low activity of CTLA-4 and CTLA-4dim, as defined for obtaining this simulation, so they are only transiently expressed at low levels and cannot exert their regulatory action (11).

**Figure 6:**
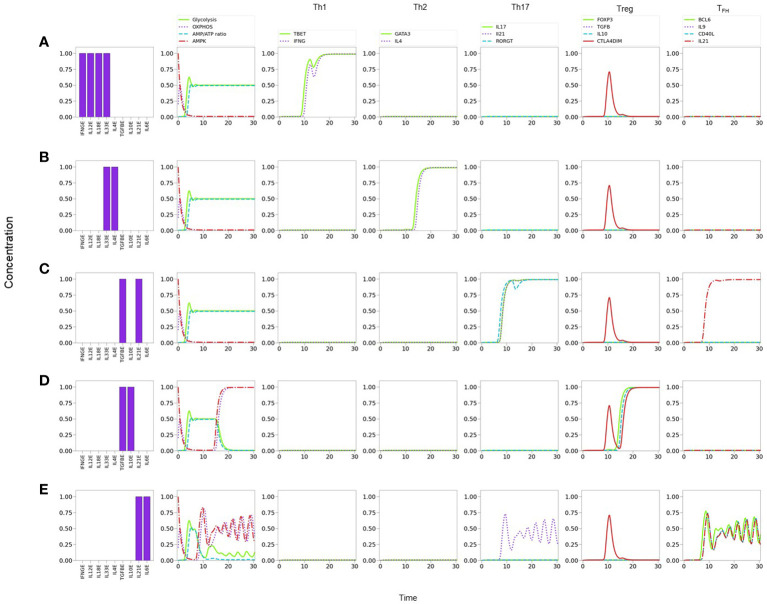
Line plots of CD4^+^ T cell differentiation and metabolic dynamics induced by specific lineage-inducing cytokine microenvironments (as indicated in histograms) and optimal nutrient and oxygen conditions. **(A)** Th1 polarization characterized by sustained expression of T-bet and IFN-*γ*. **(B)** Th2 polarization characterized by sustained expression of GATA-3 and IL-4. **(C)** Th17 polarization characterized by sustained expression of ROR*γ*T, IL-17 and IL-21. **(D)** Treg polarization characterized by sustained expression of Foxp3 and IL-10. **(E)** T _*FH*_ polarization characterized by sustained periodic expression of Bcl-6, IL-9, IL-21, and CD40L. Similarly, a periodic oxidative metabolism is also observed, preceded by a transient glycolytic phase.

**Figure 5** shows heat maps of the differentiation dynamics induced by sets of phenotype-inducing cytokines, including that associated to the T_*FH*_ phenotype, a module introduced to the model in this work. For comparison, **Figure 5A** shows the profile obtained in the absence of exogenous cytokines presented in **Figure 4**. **Figures 5B-D** correspond to Th1, Th2, and Th17 microenvironments. These conduce to a similar activation dynamics as that depicted in **Figure 5A**, including a glycoytic metabolic profile. On the other hand, Treg (**Figure 5E**) shows an initial transient glycolytic state, which afterwards is displaced by a high-level oxidative metabolism. Finally, T_*FH*_ (**Figure 5F**) exhibits a periodic metabolic pattern alternating between OXPHOS and glycolysis. In all cases, exogenous cytokines induce the expected profiles of lineage-defining transcription factors and production of cytokines: Th1, T-bet and IFN-*γ* ; Th2, GATA3 and IL-4; T_*FH*_ , ROR*γ*t, IL-17 and IL-21; Treg, Foxp3 and TGF-*β* , and T_*FH*_, Bcl-6, IL-9, IL-21, and CD40L. For clarity, the dynamics of some of the main components in the activation and differentiation processes is depicted in the form of line plots in **Figure 6**.

### 3.2 T_FH_ polarization

Notably, the model shows that T_*FH*_ cells display a periodic expression of Bcl-6, T_*FH*_ -related traits (IL-9, IL-21, and CD40L), and metabolic profile (with predominance of OXPHOS) **(** **Figures 5F**, **6E** **)**. The periodic behavior can be explained by the tight cross-regulation of transcription factors and metabolic elements involved in T_*FH*_ differentiation. By assuming optimal levels of exogenous IL-6 and IL-21 as input conditions, the model dynamics may lead to expression of either ROR*γ*t or Bcl-6, depending on the level of TGF-*β*. TGF-*β* activates ROR*γ*t, which in turn inhibits Bcl-6. Now, IL-6 also promotes the expression of the nutrient sensor AMPK, which induces MTORC2 and OXPHOS and, importantly, exhibits a negative feedback loop with MTORC1. Negative feedback loops represent self-regulatory mechanisms whose action depend on the relative expression of the constitutive components. When both expression levels are comparable, this may cause an oscillatory behavior in which each element (AMPK and MTORC1 in this case) tries to repress the other one. The oscillatory behavior may be transmitted to downstream elements in the signalling pathway **Figure 6E**. Interestingly, the oscillatory behavior of T_*FH*_ may be stabilized by lowering the levels of exogenous IL-6 and IL-21 (≤0.75). This procedure down regulates the activity of the AMPK-MTORC1 feedback loop, conducing to a stable but moderate expression of Bcl-6 and its related phenotypic traits, including the steady manifestation of mTORC1 and a glycolytic metabolism (data not shown).

### 3.3 Effector phenotype hierarchy and cytokine levels

**Figure 7:**
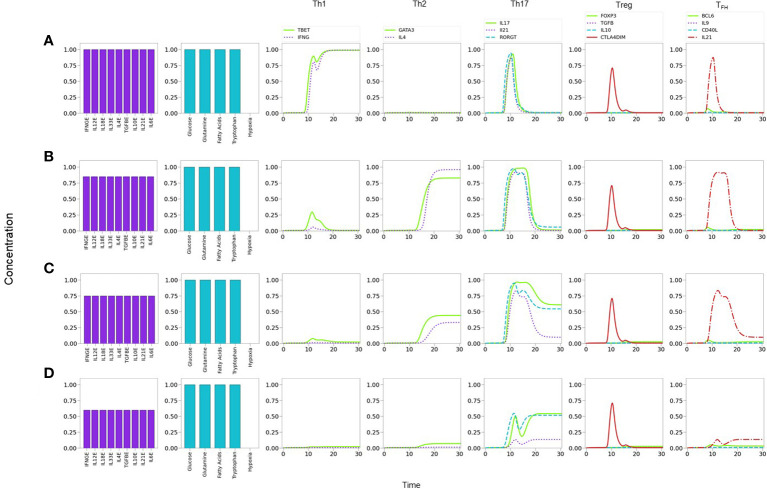
Phenotype expression in a microenvironment defined by variable levels of a uniform mixture of all-type of exogenous cytokines, under optimal nutrient and normoxic conditions (as indicated in histograms). Denoting cytokine levels by CLs, we observe that **(A)** a maximal CL = 1.0 induces a Th1 polarization, with a transient Th17 expression. **(B)** A smaller CL = 0.80 shifts the polarization towards Th2, also including a transient Th17 response. **(C)** A further decrease to CL = 0.75conduces to a mixed Th2-Th17 phenotype. **(D)** At CL = 0.6, Th2 vanishes and only Th17 is expressed at a moderate value. Eventually, at an all-cytokine threshold level CL = 0.40 no phenotype expression arises (not shown), indicating cytokine insufficiency to promote differentiation.

As a strategy to perform a systematic search of the influence of the cytokine microenvironment on the activation and differentiation dynamics, cell activation was initially simulated by assuming a background consisting of a uniform all-type mixture of exogenous cytokines (IFN-*γ* , IL-12, IL-18, IL-33, IL-4, TGF-*β* , IL-10, IL-21 and IL-6), together with optimal levels of nutrients (glutamine and tryptophan) and oxygen. It can be noticed that this kind of microenvironment could be interpreted as a cytokine storm. We then analyzed the dynamics induced by stimulation of the TCR by MHC-antigen and co-stimulation of CD28 by CD80/86, in the presence of varying levels of the all-type cytokine combination. **Figure 7** portrays the resulting expression dynamics of transcription factors and output (produced) cytokines. Denoting the cytokine level by *CL*, **Figure 7A** shows that the presence of optimal cytokine levels (*CL*=1.0) conduces to Th1 polarization, with a transient expression of the Th17 phenotype. **Figure 7B** proves that a moderate cytokine depletion (*CL*=0.80) shifts the polarization towards Th2, also including a transient Th17 response. **Figure 7C** implies that a further cytokine decrease (*CL*=0.75) induces a mixed Th2-Th17 phenotyspe, and **Figure 7D** shows that an even lower cytokine level (*CL*=0.60) yields a low expression of the Th17 phenotype. At stages associated to intermediate cytokine levels (as described in **Figure 7C**) the depletion of either IL-6 or TGF-*β* conduces to suppression of Th17, favouring Treg in the first case, or T_*FH*_ in the second case (**Suplementary Figure 1**). As expected, at sufficiently low cytokine levels (*CL*≤0.40) no phenotype is expressed (not shown).

The former results suggest the occurrence of a phenotype expression hierarchy, such that optimal levels of an all-type cytokine mixture and nutrients conduce to the expression of the highly-polarized phenotypes associated to the cellular and humoral immune response (Th1 and Th2), whereas decreased cytokine levels (with optimal nutrients) allow the transit to a region where less polarized states corresponding to pro-inflammatory and regulatory immune responses (Th17, Treg, or T_*FH*_ ) may arise. The predominance of a Th1 phenotype in the tested conditions is in agreement with experimental observations (50–54). On the other hand, the existence of an inflammatory-regulatory immune response at moderate or low cytokine levels is consistent with experimental observations of diseases induced by chronic inflammation (55). A previous model by (56) also provides several mixed phenotypes determined by the cytokine microenvironment. One main difference is that the influence of TGF-*β* is determinant in the model, so that mixed phenotypes always involve the expression of Foxp3; accordingly, a mixed T-bet-GATA3 expression is only possible when TGF-*β* is absent, in contrast with the Th1-Th2 cytokine predominance discussed here. In addition, the Puniya, et al. model predicts a mixed phenotype including the expression of GATA3 even if IL-4 is not present, which does not arise in our analysis. On the other hand, both models agree that in the absence of IL-4, the presence of Th1-inducer cytokines along with TGF-*β*, or the combination TGF-*β* -IL6, allows the respective expression of the T-bet-Foxp3, or T-bet-Foxp3-ROR*γ*t combinations. A more detailed analysis of the functional cytokine hierarchy on CD4^+^ T-cell differentiation will be presented in a forthcoming paper.

### 3.4 Effect of variable nutrient microenvironments on CD4^+^ T cell differentiation

In order to examine the effect of suboptimal levels of nutrients and oxygen on cell differentiation, we simulated the activation and differentiation of CD4^+^ T cells in a microenvironment containing optimal levels of all-type exogenous cytokines. Since many alternatives may be considered, we only report results arising from variations of glutamine (GLN), tryptophane (TRP), and hypoxia (HYP), at constant and optimal levels of glucose and fatty acid concentrations.

#### 3.4.1 Glutamine and tryptophan support Th1 and restriction leads to a Th2 phenotype

**Figure 8:**
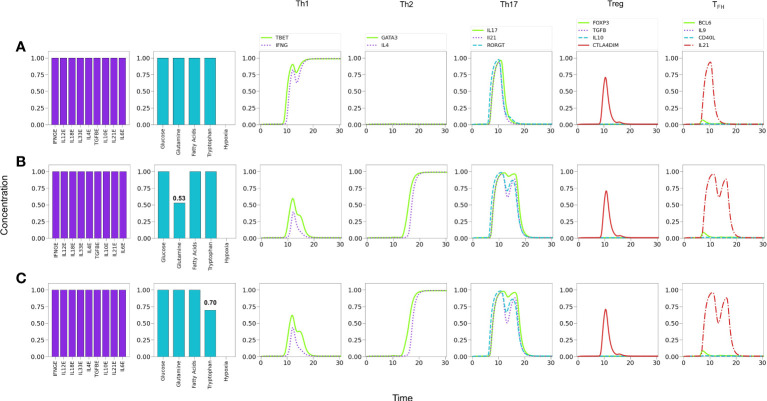
Effect of optimal and suboptimal levels of glutamine (GLN) and tryptophane (TRP) on CD4^+^ T cell differentiation dynamics in the presence of an all-type mixture of exogenous cytokines. **(A)** Optimal levels of GLN and TRP induce a Th1 phenotype, with transient expression of the Th17 profile and CTLA-4. **(B)** GLN deficiency perturbs Th1 expression; at a critical level GLN = 0.53 a Th2 phenotype is promoted, while Th1 is inhibited. The transient expression of Th17 and T _*FH*_ traits increases with respect to the first case. **(C)** TRP deficiency also perturbs Th1 expression; at a critical level GLN = 0.70 a Th2 phenotype is promoted, while Th1 is inhibited. As before, the transient expression of Th17 and T_*FH*_ traits increases. It can be observed that deficiency of GLN or TRP induces a similar differentiation dynamics, with a higher sensitivity to reduced TRP levels.

It is known that glutaminolysis reinforces the glycolytic metabolism by the production of AKG and activation of mTORC1 (8, 24), thus favoring Th1 and Th17 differentiation (28). We assessed in **Figure 8** the influence of nutrient deficiency at the set of activation in the presence of optimal levels of an all-type mixture of cytokines. As before, **Figure 8A** indicates that optimal cytokine levels (*CL*=1.0) induce Th1 polarization. Instead, restriction of glutamine induces polarization towards Th2, along with a transient but prominent Th17 profile **Figure 8B**. As shown in **Figure 8C**, tryptophan deficiency resulted in a similar restriction of the Th1 phenotype and polarization towards Th2, with a transient but conspicuous display of the Th17 profile. This observation is in agreement with data showing that tryptophan is necessary of the induction of Th1 and is important for Th17 differentiation (33, 34). Together these results suggest that Th1 is the predominant phenotype in the presence of a mixture of cytokines and this predominance can be disrupted by glutamine or tryptophan deficiency, leading cells towards alternative effector phenotypes. It can be observed in **Figure 8** that deficiency of either glutamine or tryptophan yields a very similar differentiation dynamics, although with a higher sensitivity to reduced tryptophan levels.

#### 3.4.2 In the absence of Th2-inducing cytokines, tryptophan or glutamine restriction promote Treg/Th17 phenotypes

**Figure 9:**
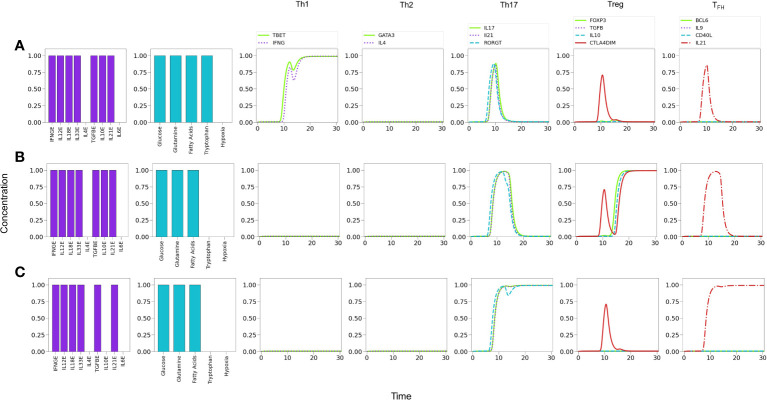
Effects of tryptophan deficiency in a microenvironment defined by Th1-, Th17- and Treg-inducing cytokines in absence of exogenous IL-4 and IL-6. **(A)** Optimal tryptophan levels induce a Th1 phenotype, with transient expression of Th17 traits. **(B)** Tryptophan deficiency inhibits Th1 expression, allowing the emergence of Treg polarization due to the activity of exogenous TGF-*β* and IL-10. An increased transient expression of Th17 is also observed. **(C)** Tryptophan deficiency, along with the absence of exogenous IL-10, depletes Treg and allows steady Th17 polarization due to the action of IL-21.

The influence of tryptophan deficiency on the differentiation dynamics was analyzed by assuming absence of IL-4 since, as noted above, optimal levels of Th1- and Th2-type cytokines strongly polarize the system towards these subtypes, impeding the expression of less-polarized phenotypes. We depict in **Figure 9** the effects of tryptophan deficiency in a background defined by Th1, Th17 and Treg exogenous cytokines. **Figure 9A** reveals that an optimal tryptophan level induces a Th1 phenotype, with transient expression of Th17. In contrast, tryptophan deficiency reduces the Th1 polarization, allowing the expression of a Treg phenotype, including a transient expression of Th17 **Figure 9B**. In these conditions, additional insufficiency of the Treg-inducing cytokine (IL-10) promotes a Th17 polarization. **Figure 9C**.

Similarly as tryptophan, glutamine induces the expression of both T-bet and ROR*γ*t; however, its action involves a larger influence in the differentiation process, as it also promotes MTORc1 activation mediated by glutaminolysis and AKG (**Figures 2**, **3**). In this case, we prefer to present our results as **Table 1**, which displays CD4^+^ T cell phenotypes arising from glutamine deficiency, including a number of hybrid phenotypes induced by specific concentrations of exogenous cytokines. Several of the hybrid phenotypes displayed in **Table 1**, like Th1/Th17, Th2/Th17, or Th17/Treg have been implicated in the development of autoimmune diseases [reviewed in (2)] and cancer (57, 58).

**Table 1:** Phenotype profiles induced by deficient glutamine levels and/or hypoxic conditions (and optimal tryptophan levels) as function of the relative concentrations of phenotype-inducing cytokines.

	GLN	Hyp	Th1	IL-4	IL-6/IL-21	TGF-*β*	IL-10	Expressed profiles
1)	1	0	1	1	1	1	1	**T-bet, IFN-** *γ*
2)	0.50	0	1	1	1	1	1	**GATA3, IL-4**
3)	0.50	0	1	1	0.65	1	1	**GATA3, IL-4 /** Foxp3
4)	0.50	0	1	0.70	1	1	1	**GATA3, IL-4 / ROR** *γ* **t, IL-17**
5)	0.50	0	1	0.63	1	1	1	T-bet / GATA3 **/ ROR** *γ* **t, IL-17, IL-21**
6)	0.50	0	1	0.50	1	1	1	T-bet, IFN- *γ* **/ ROR** *γ* **t, IL-17, IL-21**
7)	0.00	0	1	0	0.71	0	1	**ROR** *γ* **t, IL-17, IL-21/** Foxp3
8)	0.40	0.50	1	0.50	0.30	1	1	**ROR** *γ* **t,IL-17 /** Foxp3
9)	0.50	0.50	1	0.50	1	0	1	T-bet **/** ROR *γ* t, IL-17, **IL-21 / Bcl6**
10)	0.50	1	1	0.50	1	0	1	T-bet, IFN-*γ* **/ ROR** *γ* **t, IL-17, IL-21**
11)	0.50	1	1	0.50	0.30	1	1	T-bet, IFN-*γ* **/ ROR** *γ* **t, IL-17**

Each profile involves the stable expression of single or mixed transcription factors and produced cytokines. Boldface characters indicate the prevailing expression in a hybrid subtype. The Th1 column represents the joint action of IL-12/IFN-*γ* /IL-18/IL-33).

#### 3.4.3 Glutamine restriction leads to Treg in the absence of IL-4 and hypoxia promotes Th17

**Figure 10:**
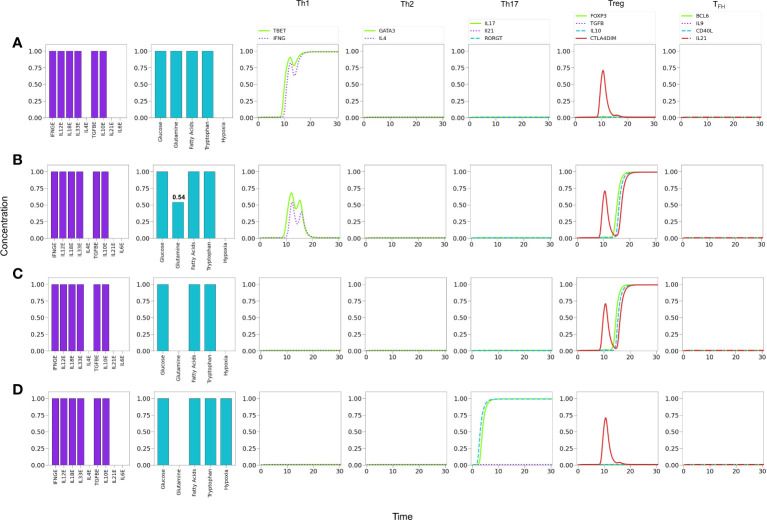
Effects of suboptimal glutamine (GLN) levels and oxygen deficiency in a microenvironment defined by Th1 and Treg exogenous cytokines. **(A)** Optimal levels of GLN without hypoxia induces a Th1 phenotype. **(B)** GLN deficiency in absence of hypoxia perturbs Th1 expression; at a critical level, *GLN*≤0.54, Th1 is inhibited allowing the expression of a Treg phenotype. **(C)** Total absence of GLN implies a complete inhibition of Th1 and predominance of Treg polarization. **(D)** Absence of GLN along with high-level hypoxia inhibits Th1 and Treg expression, promoting Th17 polarization even in absence of the pro-inflammatory cytokines IL-6 and IL-21.

It has been reported that low concentrations of glutamine in the medium promotes the induction of the Treg phenotype (8). To assess this possibility we simulated cytokine conditions where only Th1 and Treg cytokines coexist and analyzed the effect of glutamine levels **Figure 10**. **Figure 10A** shows that optimal glutamine levels induce a Th1 phenotype, whereas sub-optimal or null levels reduce the Th1 profile to a low transient or no expression, respectively, while inducing a stable Treg phenotype **Figures 10B, C**, in agreement with several reports (26, 28). Thus, the model supports experimental observations regarding the induction of Th1 cells in glutamine deficiency conditions (8, 59), provided that Th1 and Treg-inducing cytokines are present.

It has been also suggested that hypoxia promotes Th1 and Th17 phenotypes and inhibits regulatory activities (60). We explored this possibility by simulating an hypoxic condition under conditions described above (null glutamine availability). **Figure 10D** shows that hypoxia can indeed disrupt the Treg differentiation induced by low glutamine and polarizes the phenotype towards Th17 (expression of ROR*γ*t and production of IL-17). The production of IL-21 is not induced under these conditions because the need of exogenous IL-21 and IL-6 to fully activate ROR*γ*t.

#### 3.4.4 Hypoxia inhibits Treg and favours Th1/Th17 responses in low IFN-*γ* conditions

**Figure 11:**
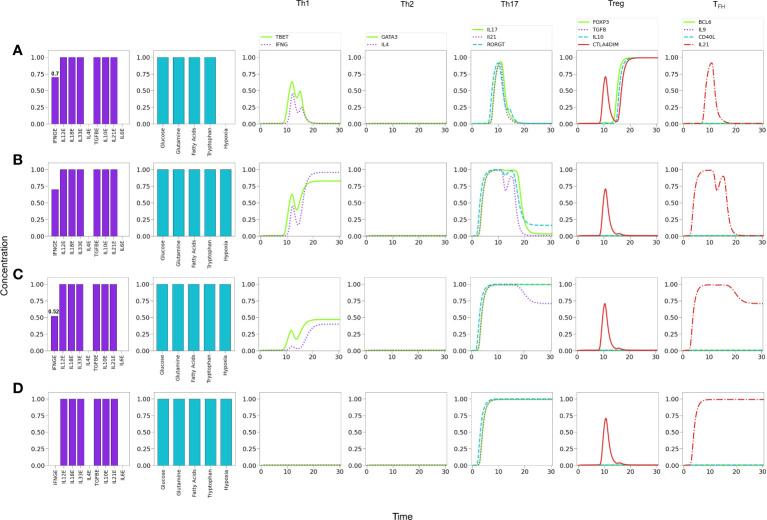
Effects of IFN-*γ* and oxygen deficiency in a microenvironment defined by Th1-, Th17-, and Treg-inducing cytokines. **(A)** Depletion of IFN-*γ* in absence of hypoxia inhibits Th1 expression and induces a Treg phenotype at a critical value IFN-*γ* = 0.7. A transient expression of Th17 is also observed. **(B)** In the former conditions (IFN-*γ* = 0.7), the action of hypoxia re-boosts Th1 and downregulates Treg, inducing a low-level expression of Th17. **(C)** A further decrease of IFN−*γ* (0.52) in conjunction with hypoxia downregulates Th1 polarization and potentiates Th17 expression. **(D)** Null levels of IFN-*γ* together with hypoxia leads to total elimination of the Th1 phenotype and a robust expression of Th17.

**Figure 11A** shows that under relatively reduced levels of IFN-*γ* , a mixture of Th1, Th17 and Treg-inducing cytokines induces predominantly a Treg phenotype. In these conditions hypoxia is able to induce a Th1 response, with a transient but clear expression of the Th17 phenotype (ROR*γ*t, IL-17 and IL-21 expression) **Figure 11B**. In order to examine the possibility of co-expression of the Th1 and Th17 phenotypes, the level of exogenous IFN-*γ* was further diminished. **Figure 11C** shows that hypoxia induces a stable Th17 phenotype and a reduced but stable Th1 phenotype. In the absence of IFN-*γ* , polarization towards Th17 is complete **Figure 11D**. Thus, both Th1 and Th17 phenotypes can coexist under hypoxic conditions at particular levels of cytokines, *e.g.*, suboptimal IFN-*γ* in absence of IL-4 and IL-6 (32, 60–62). The effect of diminution of exogenous IFN-*γ* is expected since it activates T-bet, which in turn inhibits the activity of ROR*γ*t. On the other hand, in this particular case, the Th17 phenotype is favored by the stimulatory activity of TGF-*β* and HIF-1*α* on ROR*γ*t. The effect of TGF-*β* on the regulation of the Th17/Treg balance has been addressed experimentally by (63). The model reproduces this shift under moderate hypoxia conditions (**Supplementary Figure 2**).

## 4 Discussion

Lineage-defining transcription factors tightly regulate each other upon T cell activation, so that a dynamic balance between them, as well as intrinsic metabolic changes, determines the function of differentiated CD4^+^ T cells. Added to this complexity, nutrient availability and oxygen levels are able to modulate cell differentiation. Mathematical modeling based on current experimental information provides a framework for integrating the interactions between the system components, and support the understanding of the mechanisms involved in determination of cell fate. Thus, the present model represents a conceptual scheme aimed to describe the early steps in CD4^+^ T cell activation and differentiation, taking into account the best known factors involved **(** **Figures 4**, **5** **)** and its modular structure allows the incorporation of additional elements, *e.g.*, other cytokines, signaling through nuclear receptors, etc., which may lead to a deeper understanding of T cell function. Here, we present representative results of modeling the influence of variable levels of exogenous cytokines as well as glutamine, tryptophan and oxygen, on differentiation dynamics. Notably, modeling suggests an underlying hierarchy in the development of effector phenotypes, in which the presence of optimal levels of an all-type mixture of exogenous cytokines (simulating a cytokine storm) and nutrients in the microenvironment conduces to the highly-polarized expression of phenotypes associated to cellular and humoral immune responses (Th1 and Th2), while moderate cytokine levels favour less-polarized inflammatory and regulatory responses (Th1, Treg, or T_*FH*_ ). The model also shows that the relative abundance of lineage-defining cytokines and nutrients may favor the generation of hybrid phenotypes **(** **Figures 7**-**9**, **11** and **Table 1** **)**.

The current regulatory network incorporates the induction of the T_*FH*_ phenotype and the effect of nutrients and hypoxia into a previously reported model (10, 11). In order to frame it within the scope of the model, only the initial phase of T_*FH*_ differentiation (the DC phase) has been incorporated. It includes a hypothetical interaction representing the inhibition of GATA3 by Bcl-6, not yet determined experimentally; this interaction endows the reciprocal suppression of Bcl-6 and GATA3. In the model, this functional switch renders stability to phenotypic expression. This interaction could be subject of future experimental testing. The model reproduced current experimental knowledge regarding the main events of activation, regulation, and metabolic changes along with the expression of the lineage-defining transcription factor Bcl-6. Particularly, under activation though the TCR, co-stimulation, and optimal levels of IL-6 and IL-21 as input conditions, the model rendered a periodic dynamics of Bcl-6 expression, a course of metabolism skewed to OXPHOS, and production of IL-21 **(** **Figures 5**, **6** **)**.

A Th1-type inflammatory response often occurs during cytokine storms. Th1 cells produce large quantities of IFN-γ, induce delayed hypersensitivity reactions, activate macrophages, and are essential for defense against intracellular pathogens. Our simulations show that a prevailing Th1 response is induced under T cell cytokine storm conditions **(** **Figure 7** **)**; accordingly, it is linked to a glycolytic metabolism. This result agrees with both *in vivo* and *in vitro* observations (64–66). Moreover, glutamine or tryptophan restriction skews the response from Th1 towards a Th2 phenotype (**Figure 8**), in agreement with experimental reports (67). This transition is accompanied by a transient expression of the Th17 profile. Furthermore, in these conditions, the absence of IL-4 and IL-6 promotes the induction of a Treg phenotype. In addition, the absence of IL-10 allows the full expression of the Th17 phenotype **(** **Figure 9** **)**.

On the other hand, in a microenvironment defined by Th1 and Treg exogenous cytokines, glutamine restriction leads to Th1 inhibition and induces a Treg profile, as reported in (8, 59), and also in congruence with the requirement of glutamine for maintenance of the Th1 response (67, 68) **(** **Figure 10** **)**. In these conditions, modeling shows that hypoxia shifts the balance from Treg towards the Th17 phenotype, even in the absence of the pro-inflammatory cytokines IL-21 and IL-6 **(** **Figure 10D** **)**. In this particular case, the effect is related to the stimulatory activity of TGF-*β* and HIF-1*α* on ROR*γ*t. This observations are in agreement with experimental results showing that hypoxia associates with inflammatory conditions (69, 70).

Inflammatory processes frequently involve the concomitant production of pro- and anti-inflammatory cytokines (4). **Figure 11** shows predictions of the model relative to the effect of hypoxia in a mixed cytokine microenvironment constituted by both pro-inflammatory (IFN-*γ* , IL-12, IL-18, IL-33 and IL-21) and anti-inflammatory (TGF-*β* , IL-10) cytokines. Under normoxic conditions, sub-optimal levels of IFN-*γ* render a Treg phenotype **Figure 10A**. Interestingly, a transient expression of the Th1 and Th17 profiles is observed. The model predicts that hypoxia, in combination with sub-optimal levels of IFN-*γ* promotes a shift from Treg towards Th1 and Th17 profiles (**Figures 11B-D**). Notably, the level of IFN-*γ* defines the predominance of the Th1 or Th17 phenotypes, so that decreasing levels of this cytokine diminish the Th1 response while promoting the Th17 profile. As expected, the absence of IFN-*γ* leads the system to a single Th17 response. Notably, inflammation, cytokine storm (45), hypoxia and low levels of circulating IFN-*γ* (46) have been seen gathered in COVID-19 patients who developed lung fibrosis.

The model predicts the coexistence of hybrid phenotypes under particular conditions. In this regard, it has been pointed out (2) that patients with various forms of autoimmune disease, including type 1 diabetes (47, 48), multiple sclerosis (49) or juvenile arthritis (71), exhibit mixed CD4^+^ T cell phenotypes like Foxp3^+^IFN-*γ* ^+^, a pattern reproduced by the model in conditions of low glutamine and the presence of Th1 and Treg cytokines (**Figure 10B**). Expression of hybrid Th17 cell phenotypes is associated with diseases like rheumatoid arthritis or colon cancer, which involve IL‐17^+^Foxp3^+^Treg cells or RORγt/Foxp3^+^Treg cells, respectively (57, 72), a pattern generated by modeling under lack of glutamine and IL-4, combined with moderate levels of IL-6 and IL-21, as shown in line 7 of **Table 1**.

The above discussion allows us to propose that the current model encompasses the main processes involved in activation and function of CD4^+^ T cells, so it has the potential to simulate experimental and clinical outcomes produced under particular levels of cytokines, nutrients and hypoxia, encouraging the search for the completion of predictive models.

## Data availability statement

The original contributions presented in the study are included in the article/**Supplementary Material**. Further inquiries can be directed to the corresponding authors.

## Author contributions

All authors participated in the conception, design and analysis of results presented in the work. DM-M and LH contributed to the network construction. DM-M and CV designed the mathematical formalism of the model. DM-M developed the computational programs and performed most of the calculations involved in the mathematical modeling, with partial contributions of CV. All authors contributed to the writing of the manuscript.

## Funding

This work was supported by Programa de Apoyo a Proyectos de Investigación e Innovación Tecnológica of the Universidad Nacional Autónoma de México (grant number IN215820 to LH) and a postdoctoral fellowship from CONACYT (CVU number 555239 to DM). DM acknowledges partial support from Instituto de Física, Universidad Nacional Autónoma de México.

## Conflict of interest

The authors declare that the research was conducted in the absence of any commercial or financial relationships that could be construed as a potential conflict of interest.

## Publisher’s note

All claims expressed in this article are solely those of the authors and do not necessarily represent those of their affiliated organizations, or those of the publisher, the editors and the reviewers. Any product that may be evaluated in this article, or claim that may be made by its manufacturer, is not guaranteed or endorsed by the publisher.

## Supplementary material

Supplementary Figure 1 Phenotype expression in a microenvironment defined by depleted levels (0.70) of a uniform mixture of all-type of exogenous cytokines, under optimal nutrient and oxygen conditions (as indicated in histograms). **(A)** When all exogenous cytokines are present a predominant Th17 phenotype is induced. **(B)** Absence of IL-6 prompts the manifestation of the Treg phenotype. **(C)** Absence of TGF-β induces a low level TFH phenotype.

Supplementary Figure 2
**(A)** A microenvironment defined by optimal levels of exogenous TGF-β, IL-10, IL-21cytokines, normoxic conditions, and depleted levels of tryptophan leads to a Treg phenotype and transientmanifestation of Th17. **(B)** Optimal levels of IL-10, IL-21 and tryptophan, but depleted TGF-β under hypoxiaconditions induce a Treg-Th17 shift.
