# Supplementary material for: Modeling the effect of environmental cytokines, nutrient conditions and hypoxia on CD4+ T cell differentiation
Source: Front Immunol. 2022 Sep 23;13:962175. doi: 10.3389/fimmu.2022.962175 (PMC9539201; doi:10.3389/fimmu.2022.962175)
Supplement: Supplementary file 2 [file DataSheet_2.pdf]

Supplementary Material 2. Differential Equations:

```
dTCRdt = 1 / (1 + e ** (-b * (WTCR - .5))) - DTCR * TCR

dCD28dt = 1 / (1 + e ** (-b * (WCD28 - .5))) - DCD28 * CD28

dAP1dt = 1 / (1 + e ** (-b * (WAP1 - .5))) - DAP1 * AP1

dCD25dt = 1 / (1 + e ** (-b * (WCD25 - .5))) - DCD25 * CD25

dIL2Gdt = 1 / (1 + e ** (-b * (WIL2G - .5))) - DIL2G * IL2G

dIL2Edt = 1 / (1 + e ** (-b * (WIL2E - .5))) - DIL2E * IL2E

dMTORdt = 1 / (1 + e ** (-b * (WMTOR - .5))) - DMTOR * MTOR

dZAP70dt = 1 / (1 + e ** (-b * (WZAP70 - .5))) - DZAP70 * ZAP70

dSTAT5dt = 1 / (1 + e ** (-b * (WSTAT5 - .5))) - DSTAT5 * STAT5

dNFATdt = 1 / (1 + e ** (-b * (WNFAT - .5))) - DNFAT * NFAT

dNFKBdt = 1 / (1 + e ** (-b * (WNFKB - .5))) - DNFKB * NFKB

dAKTdt = 1 / (1 + e ** (-b * (WAKT - .5))) - DAKT * AKT

dCTLA4dt = 1 / (1 + e ** (-b * (WCTLA4 - .5))) - DCTLA4 * CTLA4

dCTLA4DIMdt = 1 / (1 + e ** (-b * (WCTLA4DIM - .5))) - DCTLA4DIM * CTLA4DIM

dBCL2dt = 1 / (1 + e ** (-b * (WBCL2 - .5))) - DBCL2 * BCL2

dNDRG1dt = 1 / (1 + e ** (-b * (WNDRG1 - .5))) - DNDRG1 * NDRG1

dDAGdt = 1 / (1 + e ** (-b * (WDAG - .5))) - DDAG * DAG

dSOSdt = 1 / (1 + e ** (-b * (WSOS - .5))) - DSOS * SOS

dRASGTPRdt = 1 / (1 + e ** (-b * (WRASGTPR - .5))) - DRASGTPR * RASGTPR

dLCKdt = 1 / (1 + e ** (-b * (WLCK - .5))) - DLCK * LCK

dPDK1dt = 1 / (1 + e ** (-b * (WPDK1 - .5))) - DPDK1 * PDK1

dLATdt = 1 / (1 + e ** (-b * (WLAT - .5))) - DLAT * LAT

dPLCdt = 1 / (1 + e ** (-b * (WPLC - .5))) - DPLC * PLC

dPI3Kdt = 1 / (1 + e ** (-b * (WPI3K - .5))) - DPI3K * PI3K

dPIP2dt = 1 / (1 + e ** (-b * (WPIP2 - .5))) - DPIP2 * PIP2
```

```
dPIP3dt = 1 / (1 + e ** (-b * (WPIP3 - .5))) - DPIP3 * PIP3

dIP3dt = 1 / (1 + e ** (-b * (WIP3 - .5))) - DIP3 * IP3

dCAdt = 1 / (1 + e ** (-b * (WCA - .5))) - DCA * CA

dPKCdt = 1 / (1 + e ** (-b * (WPKC - .5))) - DPKC * PKC

dTBETdt = 1 / (1 + e ** (-b * (WTBET - .5))) - DTBET * TBET

dIFNGdt = 1 / (1 + e ** (-b * (WIFNG - .5))) - DIFNG * IFNG

dGATA3dt = 1 / (1 + e ** (-b * (WGATA3 - .5))) - DGATA3 * GATA3

dIL4dt = 1 / (1 + e ** (-b * (WIL4 - .5))) - DIL4 * IL4

dFOXP3dt = 1 / (1 + e ** (-b * (WFOXP3 - .5))) - DFOXP3 * FOXP3

dIL10dt = 1 / (1 + e ** (-b * (WIL10 - .5))) - DIL10 * IL10

dTGFBdt = 1 / (1 + e ** (-b * (WTGFB - .5))) - DTGFB * TGFB

dRORGTdt = 1 / (1 + e ** (-b * (WRORGT - .5))) - DRORGT * RORGT

dIL21dt = 1 / (1 + e ** (-b * (WIL21 - .5))) - DIL21 * IL21

dIL17dt = 1 / (1 + e ** (-b * (WIL17 - .5))) - DIL17 * IL17

dBCL6dt = 1 / (1 + e ** (-b * (WBCL6 - .5))) - DBCL6 * BCL6

dIL9dt = 1 / (1 + e ** (-b * (WIL9 - .5))) - DIL9 * IL9

dCD40Ldt = 1 / (1 + e ** (-b * (WCD40L - .5))) - DCD40L * CD40L

dMTORC1dt = 1 / (1 + e ** (-b * (WMTORC1 - .5))) - DMTORC1 * MTORC1

dMTORC2dt = 1 / (1 + e ** (-b * (WMTORC2 - .5))) - DMTORC2 * MTORC2

dLKB1dt = 1 / (1 + e ** (-b * (WLKB1 - .5))) - DLKB1 * LKB1

dAMPKdt = 1 / (1 + e ** (-b * (WAMPK - .5))) - DAMPK * AMPK

dGlycolysisdt = 1 / (1 + e ** (-b * (WGlycolysis - .5))) - DGlycolysis * Glycolysis

dOXPHOSdt = 1 / (1 + e ** (-b * (WOXPHOS - .5))) - DOXPHOS * OXPHOS

dAMPATPratiodt = 1 / (1 + e ** (-b * (WAMPATPratio - .5))) - DAMPATPratio * AMPATPratio

dHIF1Adt = 1 / (1 + e ** (-b * (WHIF1A - .5))) - DHIF1A * HIF1A

dGLUTAMINOLISISdt = 1 / (1 + e ** (-b * (WGLUTAMINOLISIS - .5))) - DGLUTAMINOLISIS * GLUTAMINOLISIS

dAKGdt = 1 / (1 + e ** (-b * (WAKG - .5))) - DAKG * AKG
```
