# Supplementary figures and images for: Modeling the effect of environmental cytokines, nutrient conditions and hypoxia on CD4+ T cell differentiation
Source: Front Immunol. 2022 Sep 23;13:962175. doi: 10.3389/fimmu.2022.962175 (PMC9539201; doi:10.3389/fimmu.2022.962175)

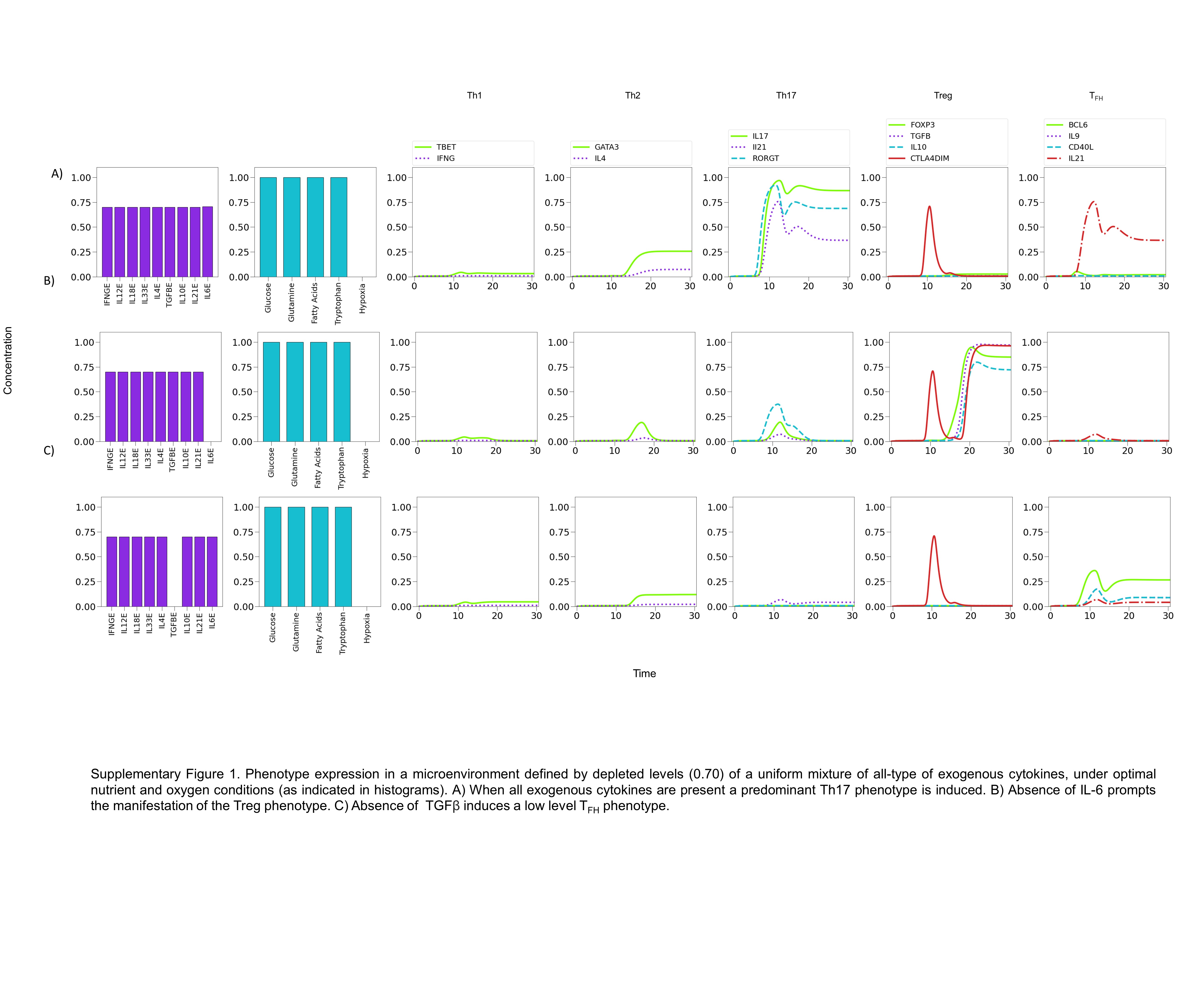

Supplement: Supplementary Figure 1 — Phenotype expression in a microenvironment defined by depleted levels (0.70) of a uniform mixture of all-type of exogenous cytokines, under optimal nutrient and oxygen conditions (as indicated in histograms). (A) When all exogenous cytokines are present a predominant Th17 phenotype is induced. (B) Absence of IL-6 prompts the manifestation of the Treg phenotype. (C) Absence of TGF-β induces a low level TFH phenotype. [file Image_1.jpg]

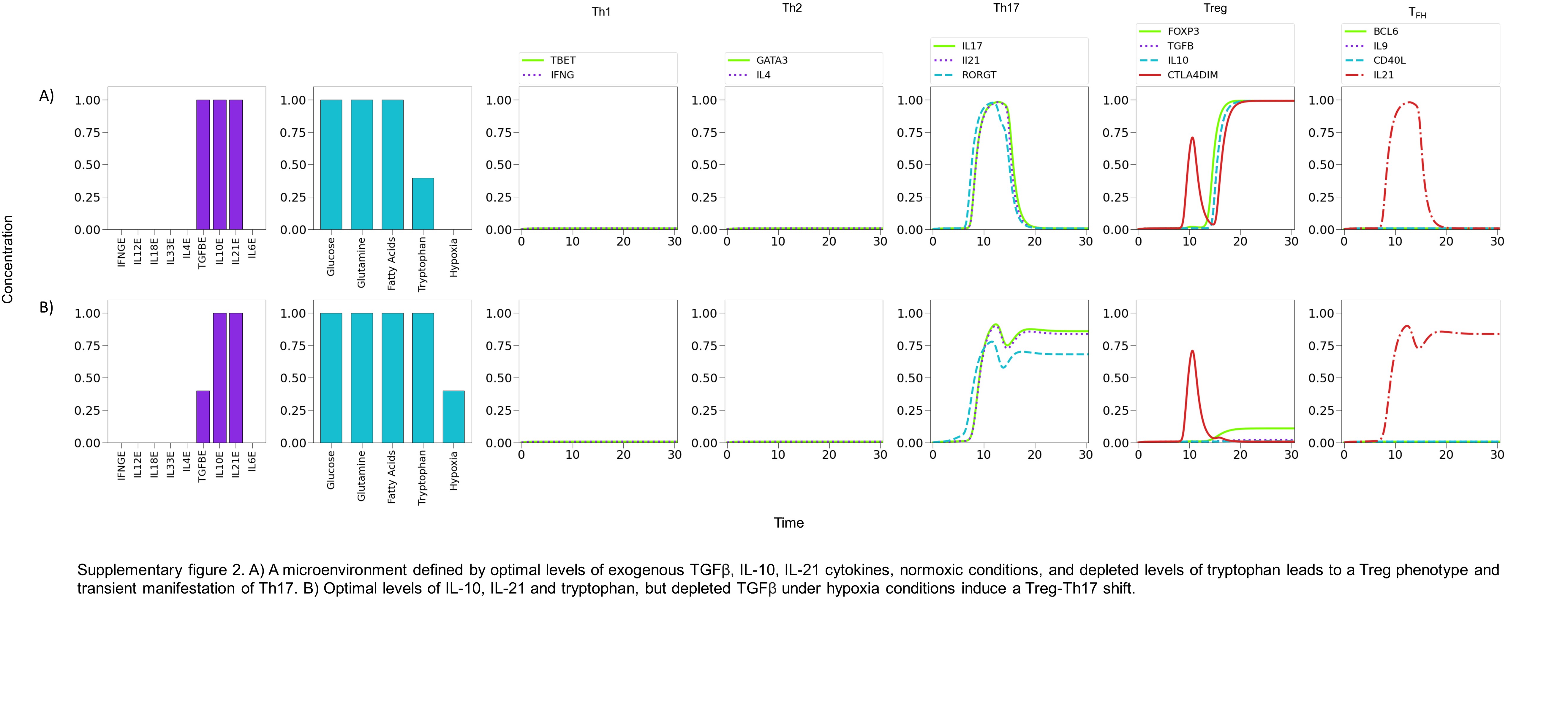

Supplement: Supplementary Figure 2 — (A) A microenvironment defined by optimal levels of exogenous TGF-β, IL-10, IL-21cytokines, normoxic conditions, and depleted levels of tryptophan leads to a Treg phenotype and transientmanifestation of Th17. (B) Optimal levels of IL-10, IL-21 and tryptophan, but depleted TGF-β under hypoxiaconditions induce a Treg-Th17 shift. [file Image_2.jpg]
